# Immediate Load‐Implant‐Supported Mandibular Advancement in a Collapsed Bite to Treat Obstructive Sleep Apnea: A Case Report

**DOI:** 10.1155/crid/1560395

**Published:** 2026-05-30

**Authors:** Maximilian Reuber, Christian Coachman, Daniel Engler-Hamm

**Affiliations:** ^1^ Dental Clinic Zahnspezialisten Theatiner, Munich, Germany; ^2^ Digital Smile Design, Madrid, Spain

**Keywords:** dental implants, digital dentistry, mandibular advancement, obstructive sleep apnea, occlusal vertical dimension

## Abstract

**Introduction:**

Patients who are edentulous or have severe loss of occlusal vertical dimension (OVD) present unique challenges in managing obstructive sleep apnea (OSA).

**Methods:**

This case report describes the full‐mouth rehabilitation of a 62‐year‐old female with an extremely collapsed bite and OSA symptoms. A digital workflow was utilized to plan a mandibular advancement prosthesis supported by dental implants. Key treatment steps included: recording a protrusive centric relation bite, increasing the OVD by 5 mm, and performing immediate implant placement with immediate loading of a provisional prosthesis. Six implants were placed in the maxilla and mandible using a guided surgical approach, and a fixed polymethyl methacrylate (PMMA) provisional prosthesis was delivered on the same day to maintain the mandible in the therapeutic forward position.

**Results:**

After 3 months of uneventful healing and osseointegration, definitive telescopic crown prostheses were fabricated as the final restorations. Function and aesthetics were fully restored, and the patient reported marked improvement in snoring and sleep quality.

**Conclusion:**

This report demonstrates that an implant‐supported mandibular advancement, combined with OVD restoration and a digital workflow, can successfully rehabilitate a collapsed bite while concurrently alleviating OSA symptoms. The interdisciplinary approach highlights the expanding role of prosthetic driven implant therapy in managing complex cases at the interface of dentistry and sleep medicine.

**Summary**

• Implant‐supported mandibular advancement can serve as a viable alternative to MADs in edentulous patients with OSA.
• Increasing OVD and anteriorly repositioning the mandible through prosthetic rehabilitation may improve upper airway patency and reduce OSA symptoms.
• A fully digital workflow enables accurate surgical execution, efficient prosthetic fabrication, and immediate functional rehabilitation in complex full‐arch cases.

## 1. Introduction

Obstructive sleep apnea (OSA) is a prevalent condition characterized by recurrent upper airway collapse during sleep, leading to oxygen desaturation and fragmented sleep [1]. Craniofacial anatomy plays a significant role in OSA; in particular, mandibular retrognathia and reduced lower facial height can predispose patients to airway obstruction [2]. Management of OSA often includes continuous positive airway pressure (CPAP) therapy or oral appliances that advance the mandible [3]. Mandibular advancement devices (MADs) have proven effective, especially in mild to moderate OSA, by repositioning the jaw forward to enlarge the pharyngeal airway [4]. However, conventional MADs require a sufficient dentition for retention, posing a challenge in edentulous or partially edentulous patients.

Edentulous patients with OSA represent a therapeutic dilemma, because they cannot use standard tooth‐borne oral appliances. Current studies suggest that improvements in OSA are less likely due to increased occlusal vertical dimension (OVD) alone [5], but rather to the associated overall positional changes of the craniofacial structures. In such cases, prosthetic rehabilitation offers an opportunity not only to restore function but also to improve airway patency. Raising a severely collapsed bite can reposition the mandible and tongue, potentially reducing airway collapse. Indeed, case reports have described treating OSA in edentulous patients by combining prosthetic jaw repositioning with implant therapy [6]. Maxillomandibular advancement surgery is the gold standard for skeletal correction in severe OSA, but a less invasive approach using implants and prosthetics may be preferable in medically compromised or older patients. Since the efficacy of MADs has been shown to persist even in patients of advanced age [7], a prosthetic approach appears to be a viable alternative to surgical intervention.

**Figure 1:**
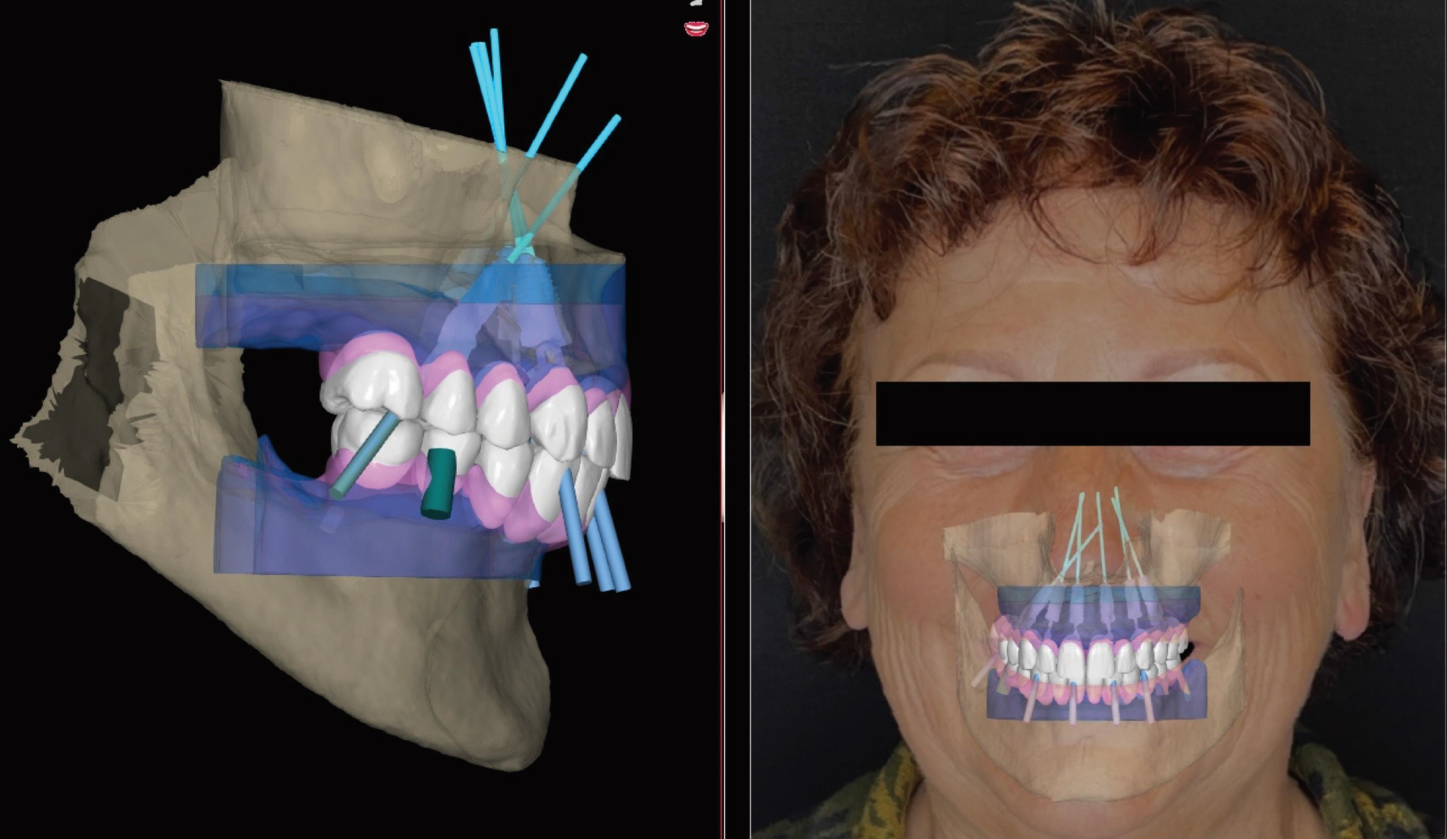
Digital workflows, incorporating cone‐beam computed tomography (CBCT), intraoral scanning, virtual treatment planning, and computer‐guided implant surgery, facilitate accurate integration of occlusal rehabilitation with implant placement.

Digital workflows, incorporating cone‐beam computed tomography (CBCT), intraoral scanning, virtual treatment planning, and computer‐guided implant surgery, facilitate accurate integration of occlusal rehabilitation with implant placement (Figure 1).

This allows clinicians to simultaneously manage OVD collapse and dental reconstruction with a high degree of precision. Immediate implant loading protocols further shorten treatment time [8], providing patients with fixed provisional teeth and improved function on the day of surgery.

This case report describes a prosthetic‐led digital implant rehabilitation with mandibular advancement, restoring function and alleviating OSA in a patient with collapsed OVD.

## 2. Case Presentation

**Figure 2:**
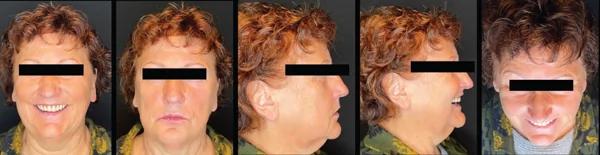
Excerpt from the DSD photo protocol. Patient full smile picture and rest position.

A 62‐year‐old female patient presented with a chief complaint of poor chewing ability and extreme jaw fatigue. She had been using outdated, ill‐fitting removable dentures in both arches for over a decade. The patient also reported chronic loud snoring and non‐restful sleep, suggestive of OSA. Her medical history was otherwise unremarkable, except for hypertension. Extraoral examination showed a reduced lower facial third due to loss of vertical dimension (Figure 2).

**Figure 3:**
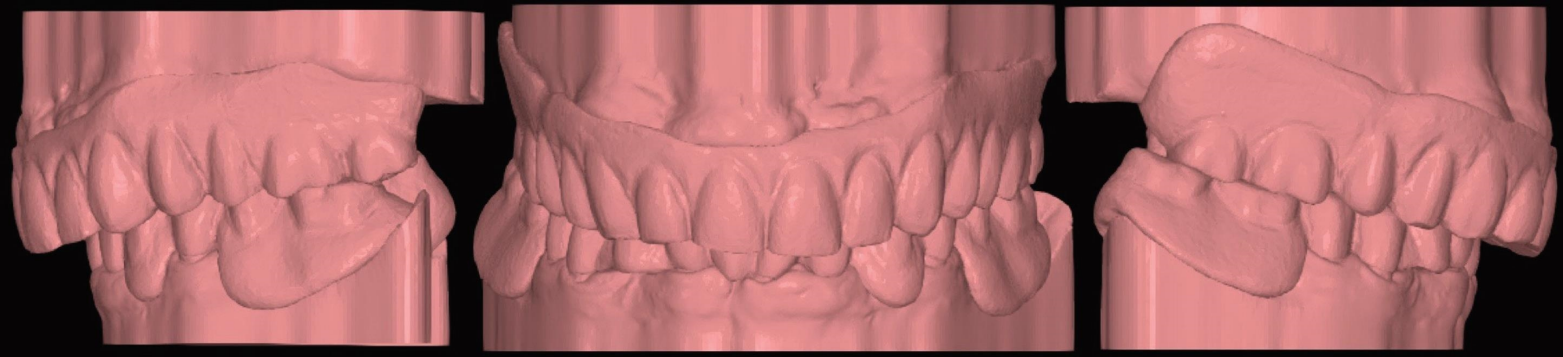
The patient presented with a severe collapsed bite but the periodontal examination revealed overall adequate periodontal health and an alveolar ridge anatomy that favored simple implant therapy.

The patient exhibited a collapsed bite: the measured freeway space was 6.5 mm, indicating a significant reduction in OVD. Intraoral exam confirmed poor fitting and worn out dentures that were anchored on few teeth with poor prognosis. The patient presented with a severe collapsed bite but the periodontal examination revealed overall adequate periodontal health and alveolar ridge anatomy that favored simple implant therapy (Figure 3).

The retruded mandible and diminished lower facial height were noted as potential contributors to her airway compromise. The patient’s Mallampati score was Class III [9] (high risk of OSA), though at presentation, she had not undergone a formal sleep study. To document the indication and later assess treatment impact, the patient completed the validated STOP‐Bang [10] questionnaire prior to therapy.

**Figure 4:**
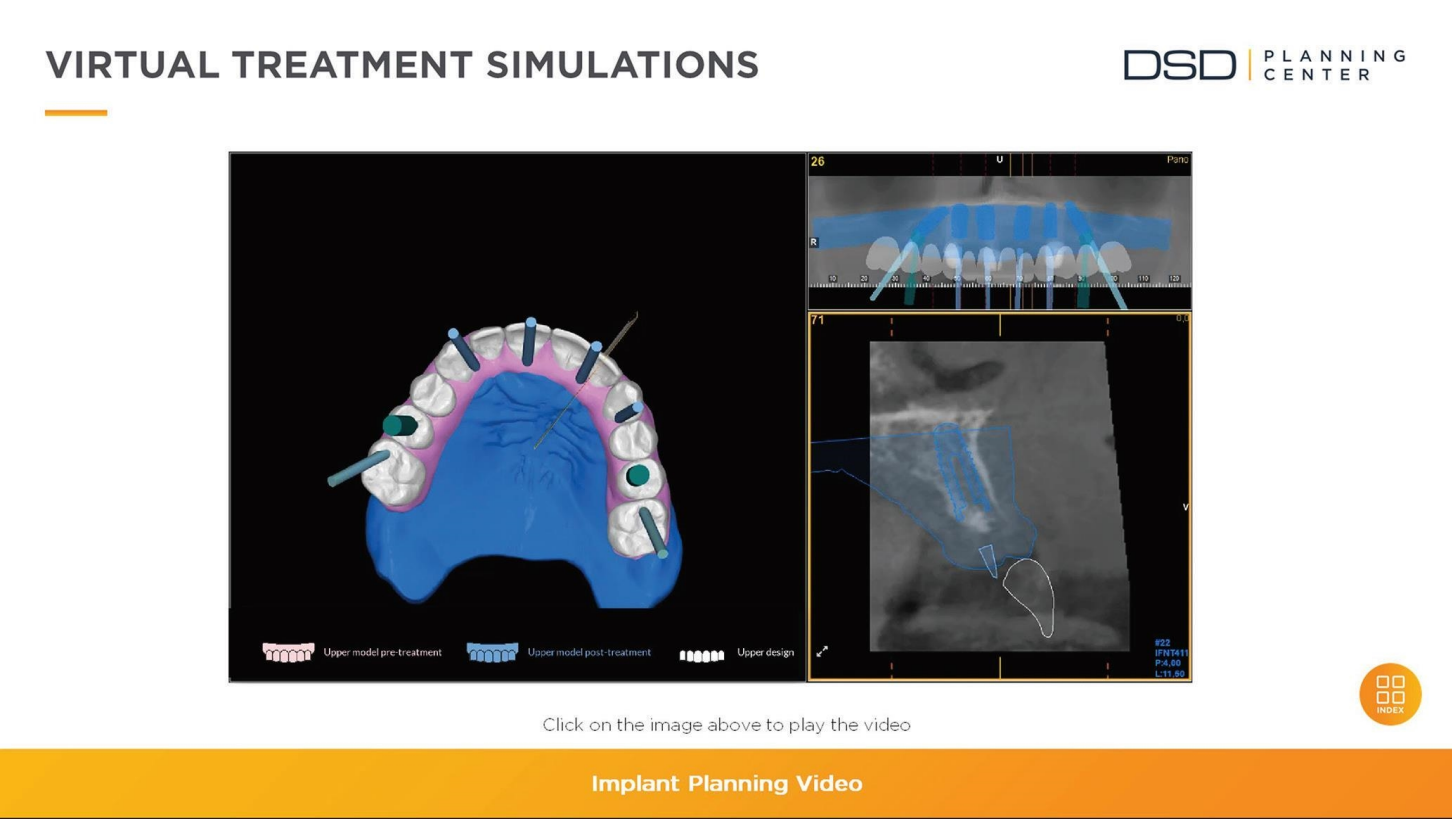
A cone‐beam CT (CBCT) scan was obtained to evaluate bone volume for implants.

**Figure 5:**
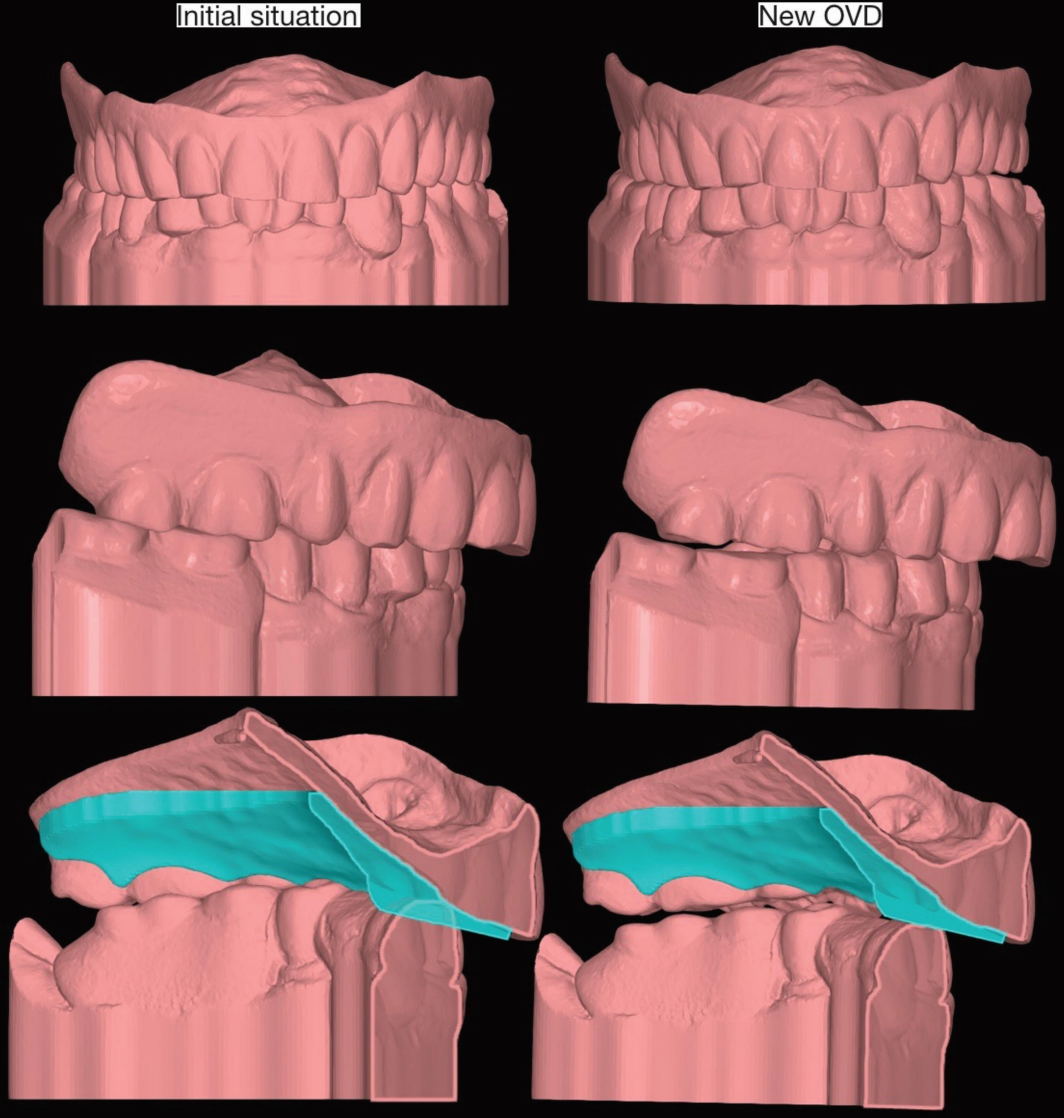
Digital study casts were taken to analyze the existing occlusion. A centric relation bite registration in a protruded position was performed using the DSD Deprogrammer.

The diagnostic workup and virtual planning were carried out in collaboration with DSD (Digital Smile Design) and a workflow was established for guided digital rehabilitation. CBCT was acquired as part of the implant planning workflow using a dental CBCT unit (Dentsply Sirona, Bensheim, Germany) with a limited field of view (FOV): Ø 8 × 8 cm/Ø 11 × 10 cm], centered on the dentoalveolar structures). The scan was used to evaluate alveolar bone anatomy and to support prosthetically driven implant positioning. Consistent with current radiographic selection principles, the limited‐FOV protocol was chosen to answer the implant‐related clinical question while minimizing radiation exposure (Figure 4). Given the patient’s edentulism and OSA symptoms, a multidisciplinary plan was developed focusing on prosthetically advancing the mandible in centric relation. Digital study casts were taken to analyze the existing occlusion. A centric relation bite registration in a protruded position was performed using the DSD Deprogrammer (Figure 5).

**Figure 6:**
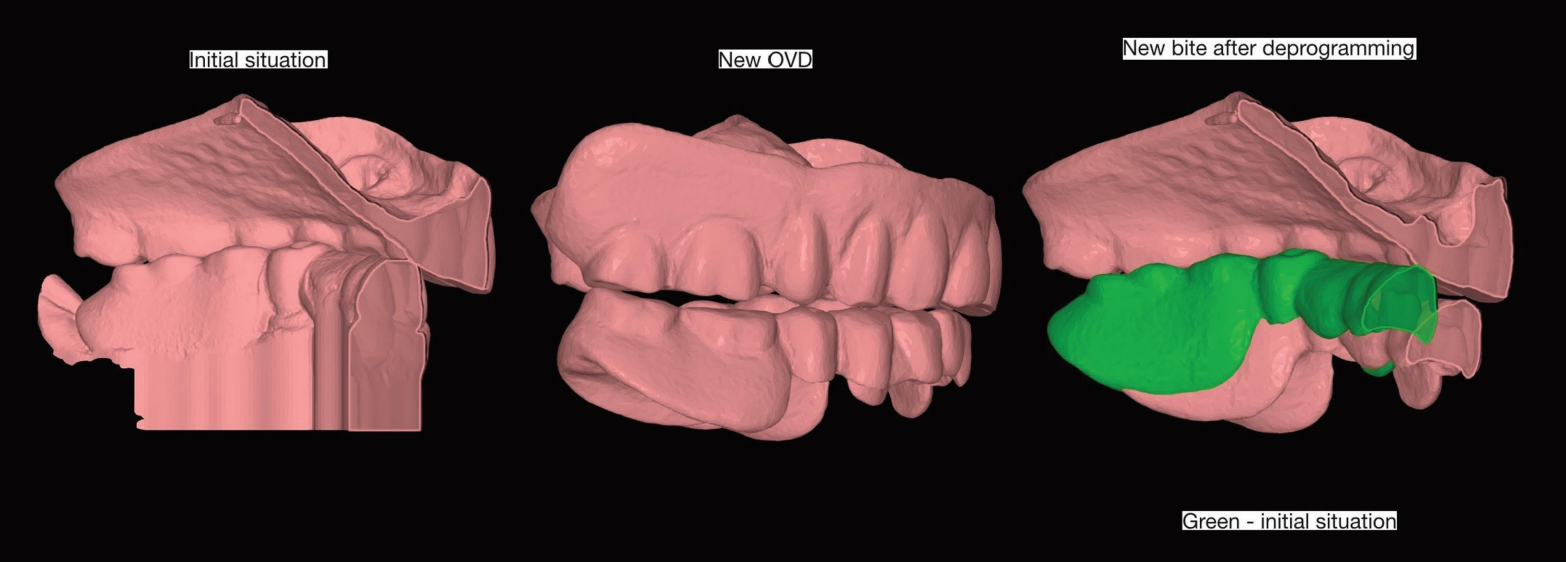
The mandible protruded without the examiner’s aid nearly to an edge‐to‐edge incisal relationship position (approximately 5–7 mm of protrusion from her habitual retruded position).

The patient wore the centric bite plate in the waiting area for 1 h and subsequently was deprogrammed with regard to her “remembered” habitual occlusion. Thereafter, her new centric relation was fixated with bite registration material. Her mandible protruded without the examiner’s aid nearly to an edge‐to‐edge incisal relationship position (approximately 5–7 mm of protrusion from her habitual retruded position) (Figure 6).

**Figure 7:**
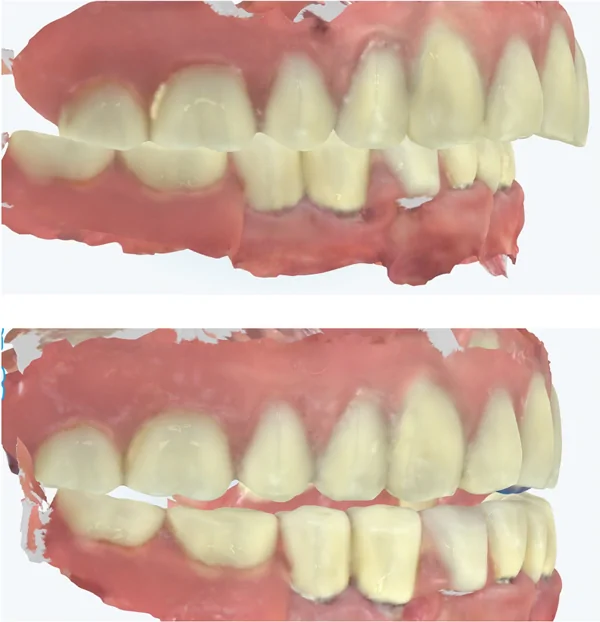
The anterior bite was chosen to optimize airway patency while maintaining a comfortable, neuromuscularly stable jaw position.

After her determined freeway space was found to be approximately 6.5 mm, it was decided to elevate her vertical bite of occlusion (VDO) 5 mm in the new centric relation position. This position was recorded digitally and would serve as the new occlusion in which treatment planning and rehabilitation subsequently was carried out. The anterior bite was chosen to optimize airway patency while maintaining a comfortable, neuromuscularly stable jaw position (Figure 7).

### 2.1. Treatment Planning

**Figure 8:**
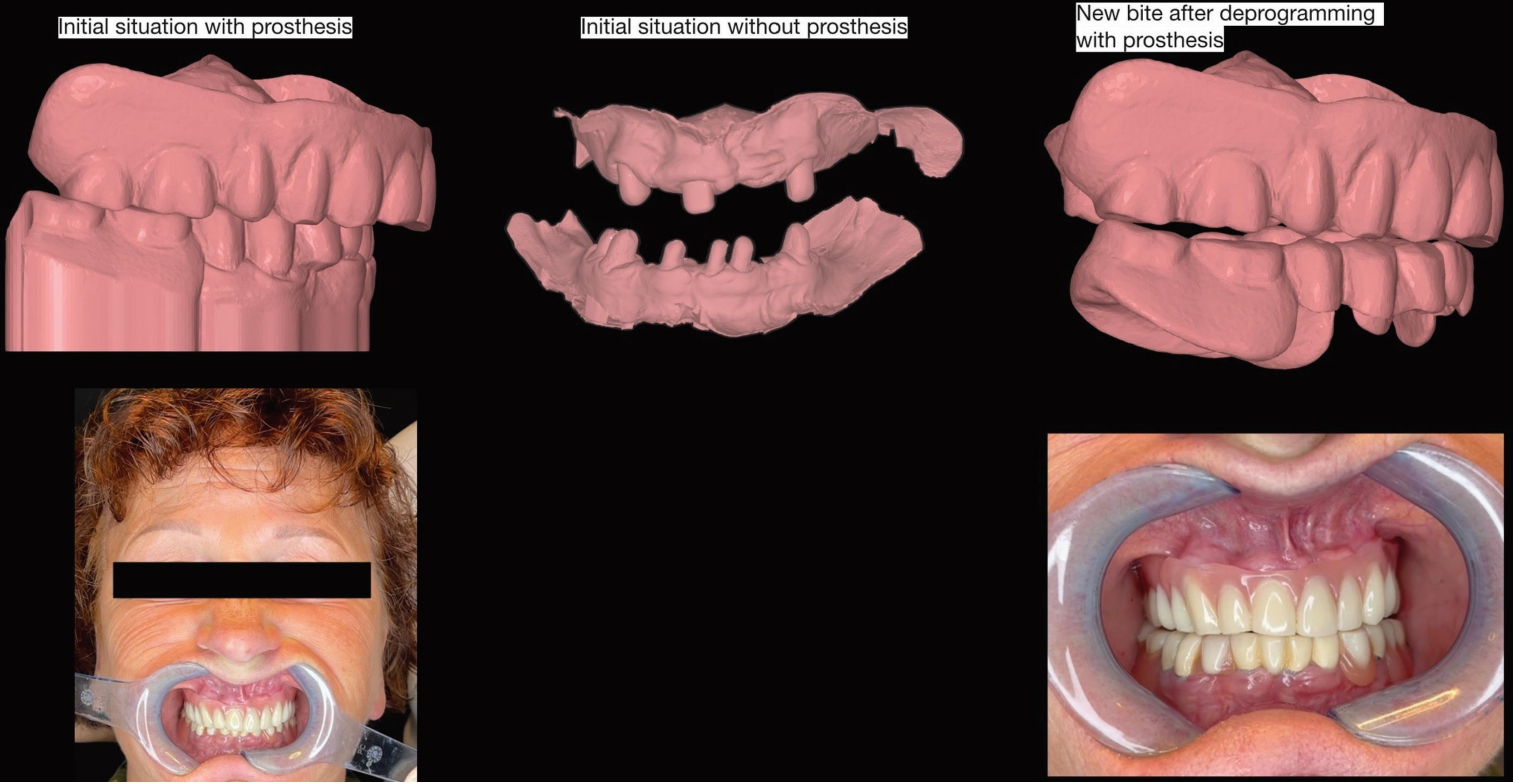
The digital scans with and without prosthesis and the third scan in the newly found bite position were recorded together with extraoral pictures and videos of the patients smile and rest positions.

**Figure 9:**
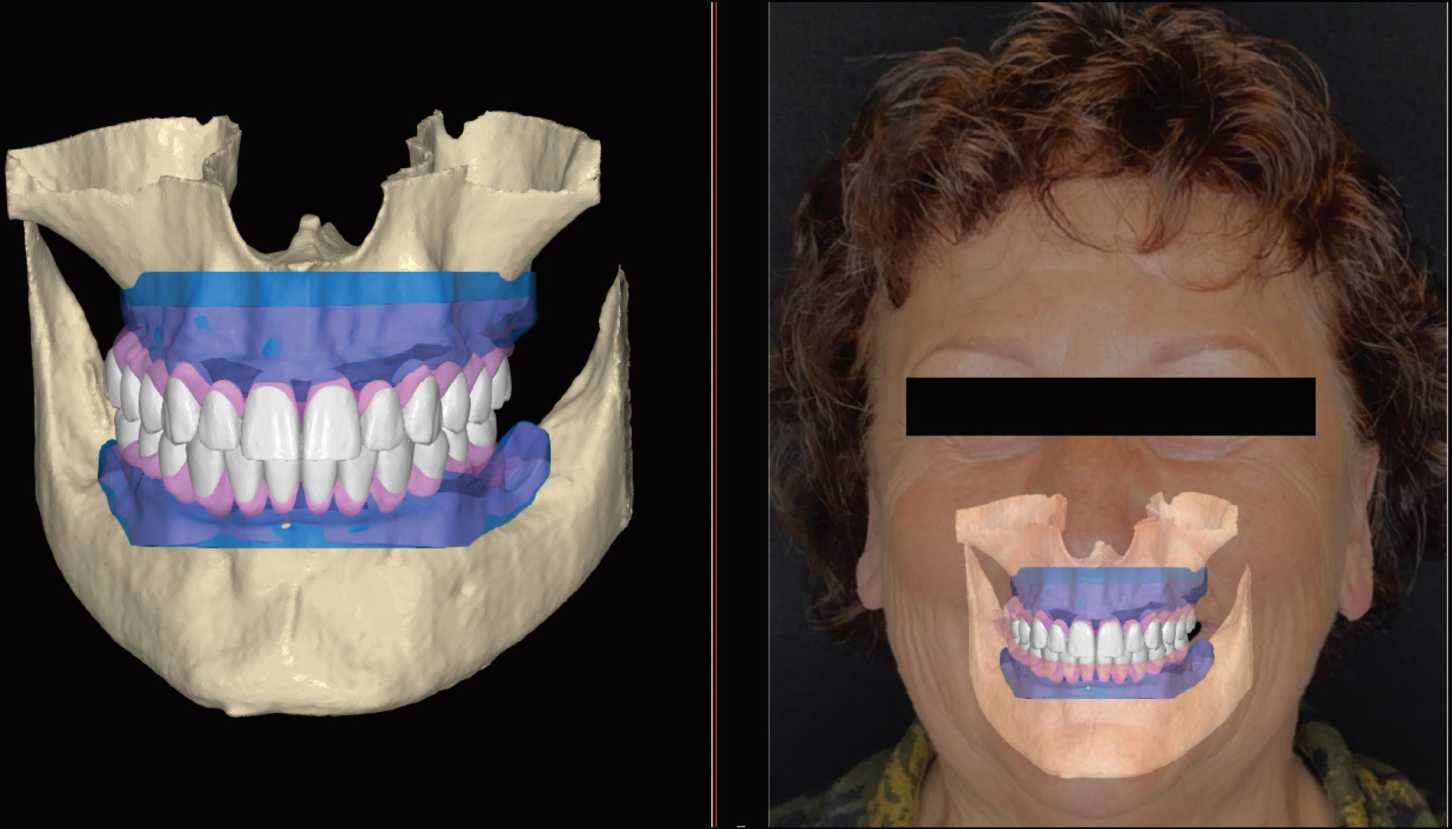
The data were matched one with the other for quality control and treatment planning purposes and additionally matched with the CBCT in the DSD Planning Center, Madrid. A virtual wax‐up was created to simulate the proposed rehabilitation in the new jaw relationship.

The digital scans with and without prosthesis and the third scan in the newly found bite position were recorded together with extraoral pictures and videos of the patient’s smile and rest positions. The data were matched one with the other for quality control and treatment planning purposes and additionally matched with the CBCT in the DSD Planning Center, Madrid. A virtual wax‐up was created to simulate the proposed rehabilitation in the new jaw relationship (Figure 8, Figure 9).

The plan had the following objectives:

1. OVD Increase: Restore approximately 5 mm of lost OVD to re‐establish a normal lower facial height, freeway space and occlusal relationship.
2. Mandibular Advancement: Reposition the mandible anteriorly to the recorded therapeutic centric protrusive position, with the aim of improving airway space.
3. Implant Placement: Immediate Extraction of all teeth and simultaneous placement of six dental implants (ZimVie Biomet Certain, FL, United States) strategically in the maxilla and mandible to support a fixed provisional and definitive prostheses. Implants were planned in a distributed manner to maximize anteroposterior spread for prosthetic support. A one‐stage fully guided surgery was designed, employing digital implant planning software (Christian Coachman, Software: Nemotec) and 3D printing of a stereolithographic surgical guide. The so‐called click guide is unique, as it is a stackable similar to the children’s Lego system. It allows all necessary guides and prosthesis’s to be stacked one into another so that the guided implant position and bite relation is transferred from a partially dentate upper and lower arch into the new fixed occlusion using a prefabricated prosthesis.

The comprehensive plan was reviewed with the patient.

### 2.2. Surgical and Prosthetic Workflow

**Figure 10:**
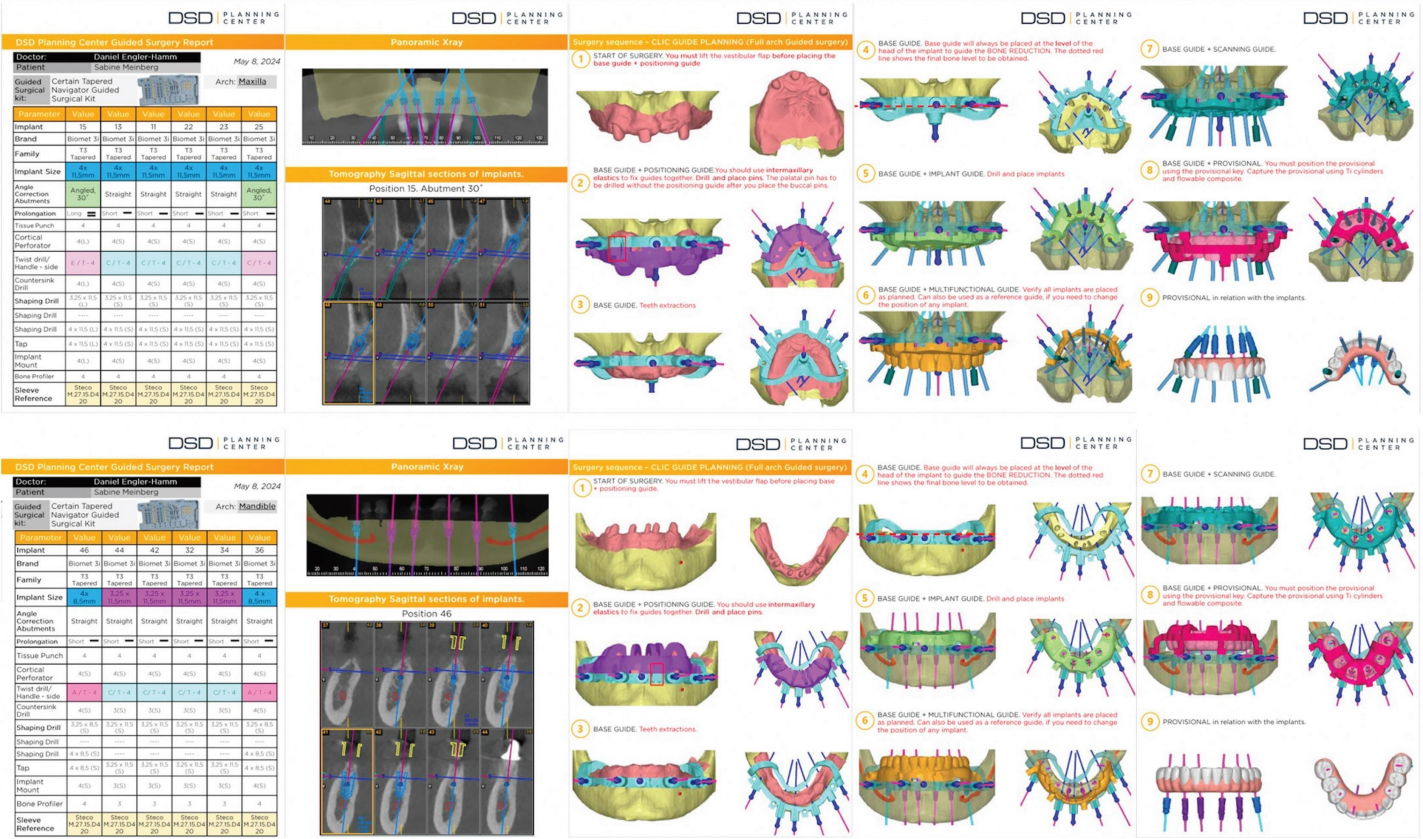
Surgical and prosthetic workflow: The procedure was performed under general anesthesia. Following ridge exposure, six implants per arch were placed using a guided surgical protocol (ZimVie, Biomet Certain, FL, United States diameter 4.1 mm, lengths 10–12 mm).

The procedure was performed under general anesthesia. Following ridge exposure, six implants per arch were placed using a guided surgical protocol (ZimVie, Biomet Certain, FL, United States diameter 4.1 mm, lengths 10–12 mm) (Figure 10).

All implants achieved a primary stability of ≥ 35 Ncm. Final positioning was confirmed radiographically. Multi‐unit abutments were attached to the implants in the maxilla to facilitate implementation of an immediate fixed prosthesis.

Immediately after implant placement, both prefabricated prosthesis were delivered and the pre‐planned protrusive jaw relationship was verified. This provisional prosthesis was anchored to the maxillary and mandibular implants on the day of surgery (immediate loading [11]). Minor adjustments of the occlusion were necessary, verified in static and dynamic occlusion using different colored indicators and corrected by minimal grinding of the prosthetic teeth. The patient left the clinic the same day with a full‐arch fixed provisional prosthesis, firmly occluding in the planned advanced position. Postoperative instructions included a soft diet and diligent oral hygiene. The patient was monitored weekly for the first month to monitor the wound healing process. She experienced mild post‐surgical discomfort but reported immediate improvement in her breathing and cessation of snoring at night. The STOP‐Bang questionnaire indicated a change from high to intermediate risk pre‐ to post‐treatment. Prior to treatment, the patient answered five items positively, corresponding to a high‐risk classification according to established STOP‐Bang criteria for case assessment. Post‐treatment, negative response to Questions 1 and 2 (snoring and daytime tiredness) reduced the total number of positive items to 3, resulting in reclassification to an intermediate‐risk category in accordance with the scoring guidelines. A change in the Mallampati classification could not be determined. By the 1‐month follow‐up, the surgical sites had healed without complication and the patient was comfortable in her new jaw position and vertical dimension.

### 2.3. Definitive Rehabilitation

**Figure 11:**
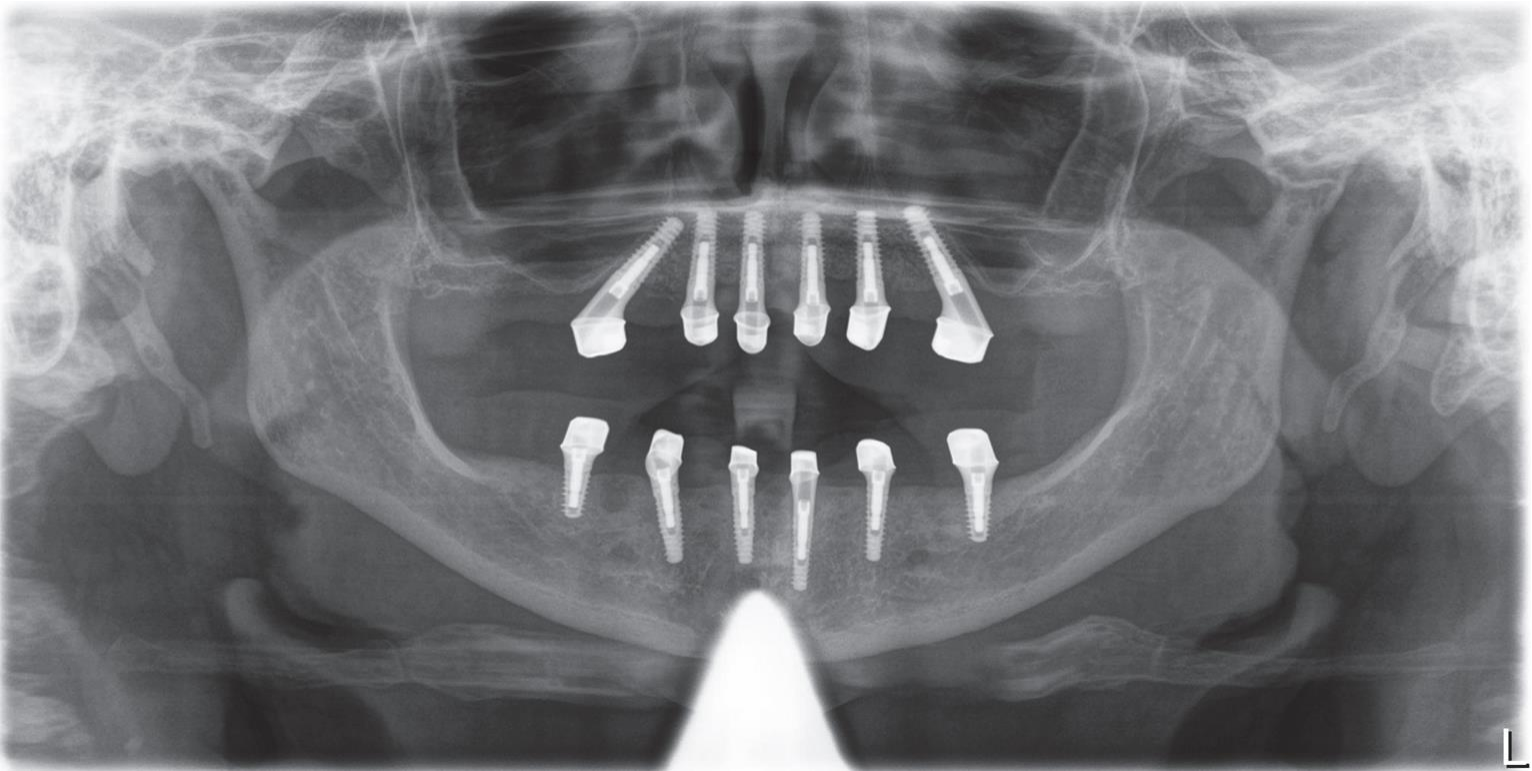
Panoramic X‐ray: After 3 months of uneventful osseointegration, implant stability was confirmed, and definitive prostheses were planned. Both arches were restored with telescopic overdentures on six implants each.

After 3 months of uneventful osseointegration, implant stability was confirmed, and definitive prostheses were planned. Three months postsurgery, regular analog implant level impressions were made and final prosthesis were delivered. Both arches were restored with telescopic overdentures on six implants each (Figure 11).

**Figure 12:**
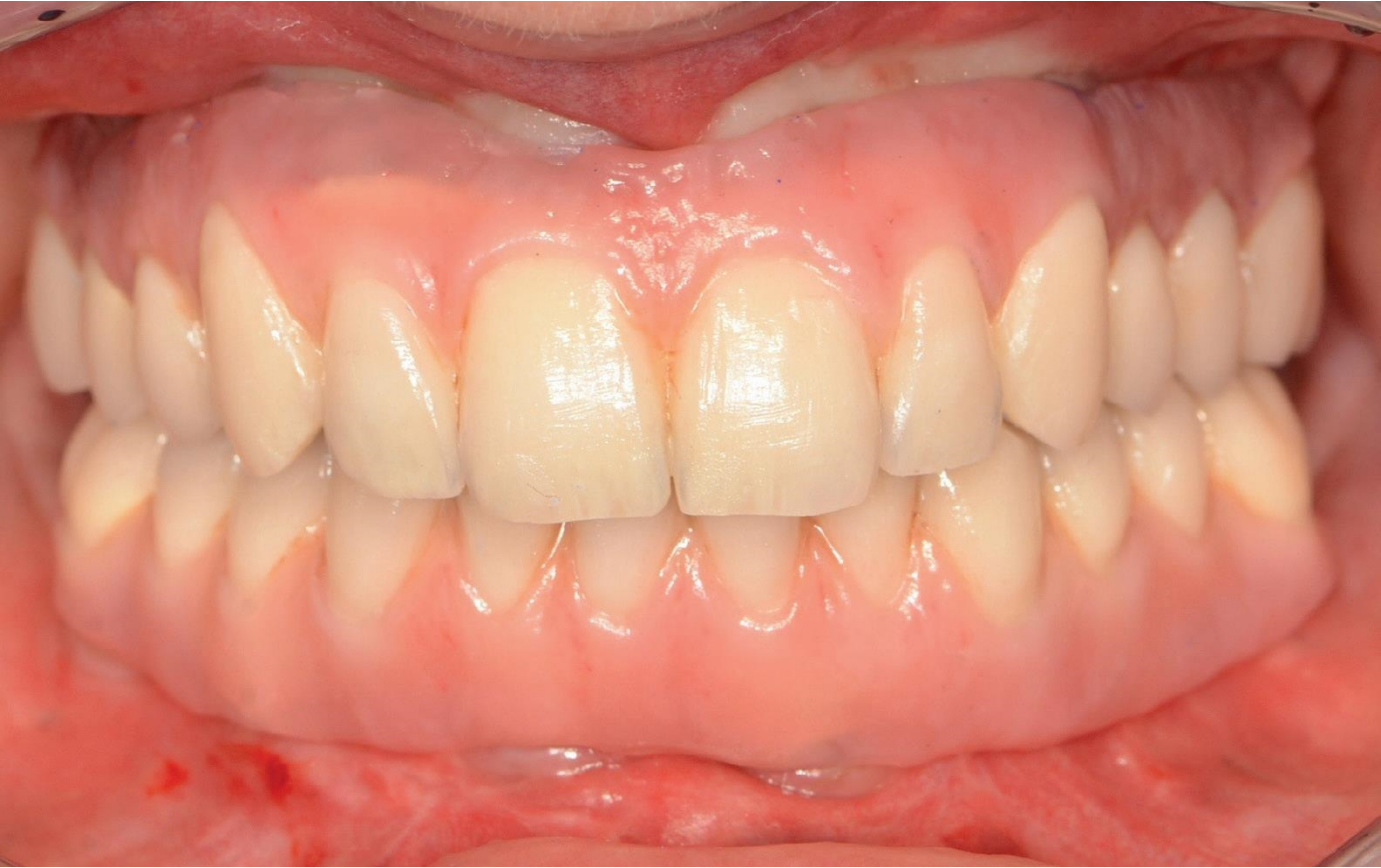
The occlusion of the definitive prostheses was set to the same OVD and jaw position as the provisional.

**Figure 13:**
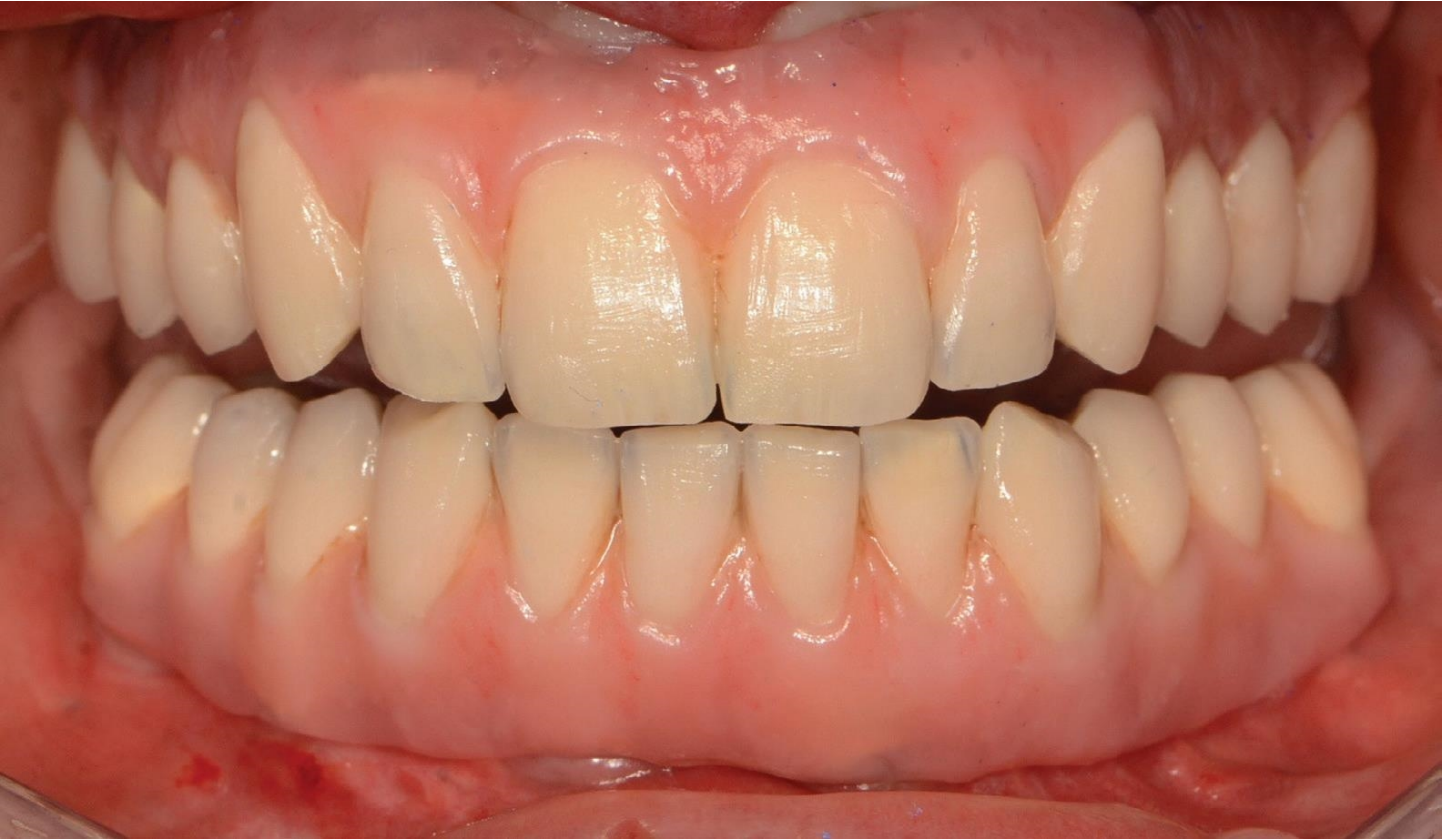
Balanced articulation to ensure stability during function.

Custom primary copings and friction‐fit secondary structures provided stability, hygiene‐friendly removability, and optimal load distribution for the resorbed ridges. The occlusion of the definitive prostheses was set to the same OVD and jaw position as the provisional (Figure 12), with balanced articulation to ensure stability during function (Figure 13).

**Figure 14:**
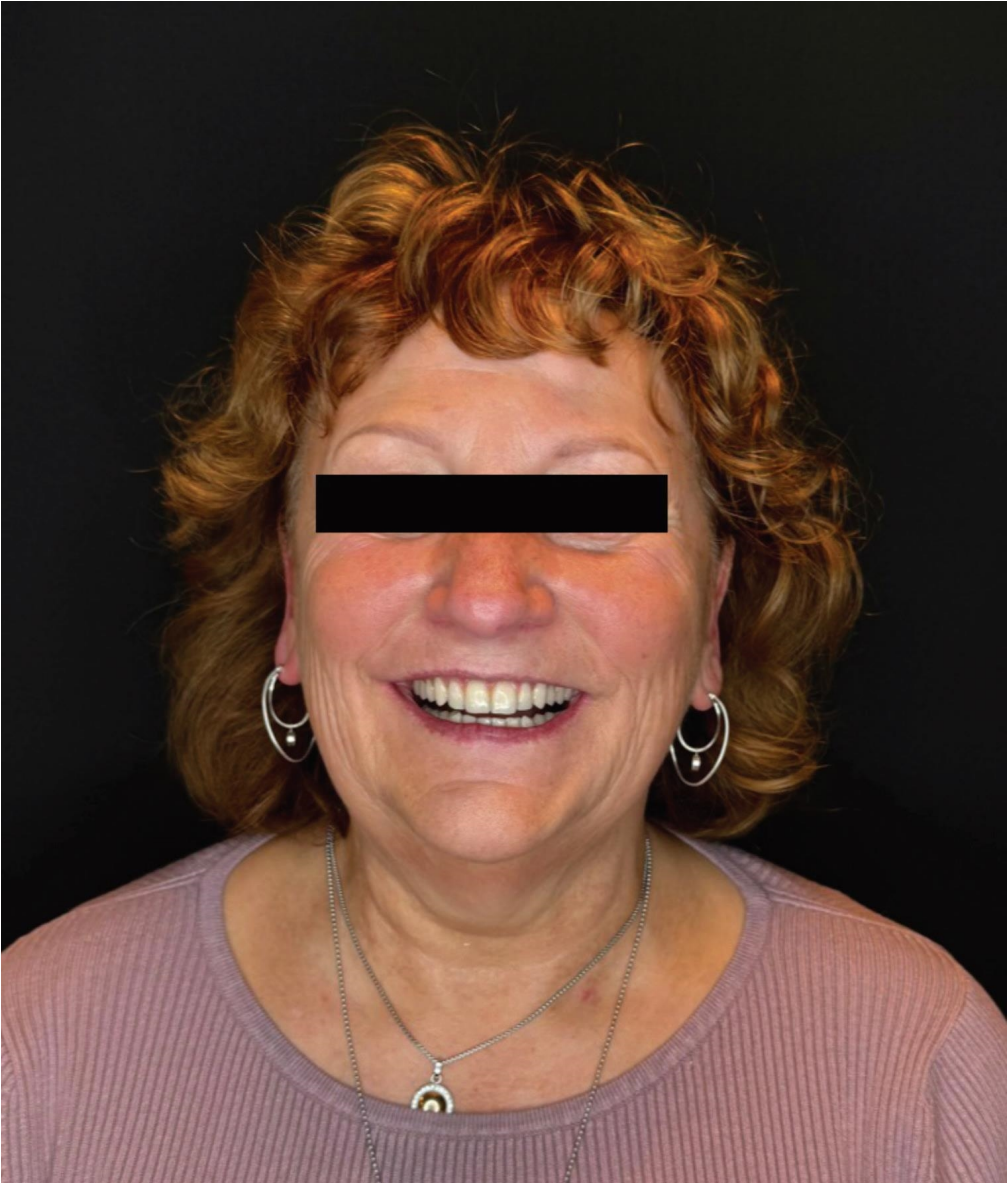
The telescopic prostheses demonstrated a harmonious occlusion and satisfactory aesthetics, restoring proper facial proportions.

The telescopic prostheses demonstrated a harmonious occlusion and satisfactory aesthetics, restoring proper facial proportions (Figure 14).

### 2.4. Outcome and Follow‐Up

The outcome was a successful full‐mouth rehabilitation that met the functional and esthetic expectations. Notably, the patient reported a dramatic improvement in her sleep quality. According to her and her spouse’s account, snoring had largely resolved with the new prostheses in place at night, and she no longer experienced daytime fatigue. While a formal post‐treatment sleep study could not be immediately obtained, these subjective improvements strongly suggest a reduction in OSA severity. At the 6‐month follow‐up, the prostheses were functioning well, peri‐implant tissues were healthy with no peri‐implantitis, and the patient maintained a high level of satisfaction.

## 3. Discussion

This case demonstrates an interdisciplinary solution to simultaneously address severe occlusal collapse, i.e., mandibular retrusion and OSA via an implant‐supported prosthetic approach. The use of a digital workflow was integral to treatment precision and efficiency. Key aspects of the case are discussed below:

1. Restoration of OVD: Reestablishing the patient’s OVD was critical for both functional rehabilitation and potential airway benefits. Although current evidence suggests that vertical bite opening alone may not be a decisive factor in the efficacy of MADs for the treatment of OSA [12], related parameters, such as the alteration of mandibular positioning achieved through prosthetic rehabilitation, play a substantial role in enhancing treatment outcomes [13]. The patient’s collapsed bite position likely contributed to a reduced oropharyngeal space. By protruding the mandible anteriorly, the mandible and tongue were repositioned inferiorly and anteriorly, which can enlarge the pharyngeal airway. Proper OVD restoration also optimizes jaw muscle function and esthetics. Importantly, studies have shown that the masticatory system can adapt to moderate increases in OVD without long‐term temporomandibular joint (TMJ) dysfunction [14]. In this case, the patient did not develop any TMJ pain or muscle issues. On the contrary, removing her overclosure relieved strain and muscular tension significantly. Thus, concerns about TMJ health should not deter clinicians from restoring a collapsed bite. In fact, restoring a severely collapsed OVD in an edentulous patient with OSA may be beneficial in two ways: it improves prosthetic function and may aid in alleviating airway obstruction. Lenganey et al. reported that in a patient with vertical and sagittal insufficiency of the lower face, increasing the lower facial height was a key part of successful OSA treatment [15].
2. Mandibular Repositioning and Airway Improvement: The anterior repositioning of the mandible in this case was designed to mirror the effect of a conventional MAD appliance, as noted in earlier observational studies [16]. Mandibular advancement has well‐documented benefits for OSA, as it tightens the soft tissues and enlarges the retroglossal space, thereby reducing airway collapsibility. Even in severe OSA cases with high baseline apnea–hypopnea indices, significant mandibular advancement (often 5–10 mm) can drastically reduce apneic events [17]. In our patient, the advancement was approximately 6 mm from her retruded position, combined with the 5 mm increase in bite height. Although we did not obtain polysomnography data, the patient’s resolution of snoring and daytime sleepiness suggests a meaningful reduction in OSA severity. This aligns with the experience of Lenganey et al. [15], who managed a similar edentulous patient with class II OSA by prosthetically advancing the mandible and subsequently performing orthognathic surgery; in their report, mandibular propulsion alone (prior to surgery) already yielded a positive, stable improvement in OSA metrics. Our case took a purely prosthetic route without orthognathic surgery, indicating that for some edentulous patients, an implant‐supported forward posture of the mandible may suffice to control OSA symptoms. It is important to emphasize the need for medical collaboration: such patients should be co‐managed with a sleep physician, and objective sleep studies are ideal to quantify improvement.
3. Guided implant surgery improves the accuracy of implant positioning and angulation [18]. This was essential to ensure correct position of the prefabricated interim prosthesis and ideal implant placement, especially for telescopic attachments. High primary stability allowed immediate loading with a fixed provisional, maintaining mandibular position and avoiding edentulism. Eini et al. found no significant difference in implant success between immediate and delayed loading in a meta‐analysis, noting that immediate loading significantly reduces treatment time without compromising outcomes [8].
4. Digital Workflow Efficiency: A fully digital workflow enabled seamless integration of surgical and prosthetic planning. Virtual setup at increased OVD and advanced mandibular position guided implant placement, reducing intraoperative risks, and enhancing treatment outcome and safety. Pre‐milled provisionals were refined chairside and delivered immediately. The precise fit of the definitive prosthesis required minimal adjustment, reflecting accurate digital calibration. Patient experience was enhanced by fewer appointments, reduced treatment costs and greater comfort. Digital workflows have been shown to reduce clinical time and improve patient outcomes in full‐arch cases [19]. In a similar report by Nowicki and Osypko, a fully digital process enabled full‐mouth implant rehabilitation with immediate loading in a periodontitis patient, and the authors concluded that a digital workflow is a reliable method even in complex cases, helping patients regain function quickly [20].

### 3.1. Challenges and Limitations

This approach, while successful, requires careful case selection and substantial expertise in prosthodontics, surgery, and digital planning. It is resource‐intensive, relying on advanced imaging, software, and milling technologies not universally available. Immediate loading depends on achieving high primary stability and mandates close monitoring. If stability had been insufficient, the treatment plan would have needed revision. A key limitation is the lack of pre‐ and post‐treatment sleep study data; subjective OSA improvement is encouraging but unconfirmed objectively.

The CBCT dataset was obtained for implant planning using a dental limited‐FOV protocol and did not include the complete upper airway. Therefore, no CBCT‐based airway volumetry, minimum cross‐sectional area assessment, or imaging‐based sleep apnea treatment planning was performed. Airway‐related considerations and follow‐up were based on clinical screening (STOP‐Bang) and the patient’s symptom report after completion of the rehabilitation, rather than on radiographic airway measurements.

As a single case, findings are not generalizable. Long‐term follow‐up is essential, and broader studies are needed to assess the role of implant‐supported mandibular advancement in managing OSA. Additional limitations include restricted titration (adjustability) of the degree of mandibular advancement and the potential for temporomandibular joint (TMJ) complications if advancement is excessive.

## 4. Conclusion

This case underscores the transformative potential of digital implant dentistry in addressing complex rehabilitation needs. By integrating thorough diagnostics, guided implant surgery, and prosthetically driven planning, simultaneous restoration of a collapsed occlusion and mitigation of OSA symptoms was achieved. The outcome exemplifies how a well‐coordinated interdisciplinary approach can extend the impact of periodontal and implant therapy beyond the oral cavity to improve overall health and quality of life. Implant‐supported mandibular advancement offers a viable treatment option for patients with OSA who cannot efficiently be rehabilitated using full or partial dentures. The use of immediate loading and digital workflows greatly enhanced treatment efficiency and precision.

We conclude that a carefully planned full‐mouth rehabilitation with OVD increase and mandibular protrusion can be successfully accomplished in one stage procedure, providing both functional rehabilitation and potential OSA relief. This approach represents a meaningful interface between periodontics, prosthodontics, and sleep medicine, focused on comprehensive patient care.

Periodontists and Periodontal Training Programs are encouraged to utilize and facilitate modern digital treatment concepts that may go beyond the scope of classic periodontal and implant therapy to enhance patient care outcome.

In the DSD planning center, there are dentists and dental specialists from various disciplines who help and jointly work together with the treatment provider to pursue patient care anywhere in the world and hereby optimize and ease treatment.

## Author Contributions

**Dr. Maximilian Reuber:** writing – original draft preparation (lead); writing – review and editing (equal); investigation (supporting); resources (supporting) **Dr. Christian Coachman:** writing – review and editing (equal); data curation (lead); investigation (equal); methodology (lead); software (supporting); visualization (lead) **Dr. Daniel Engler-Hamm:** investigation (lead); methodology (lead); validation (lead); writing – review and editing (equal).

## Funding

No funding was received for this manuscript.

## Disclosure

All authors have read and approved the final version of the manuscript. Dr. Maximilian Reuber had full access to all of the data in this study and takes complete responsibility for the integrity of the data and the accuracy of the data analysis.

## Ethics Statement

The study is an anonymized case report with informed consent obtained from the patient. Ethical approval was not required.

## Consent

Written informed consent was obtained from the patient for publication of this case report and any accompanying images.

## Conflicts of Interest

The authors declare no conflicts of interest.

## Data Availability

The data that support the findings of this study are available from the corresponding author upon reasonable request. The data are not publicly available due to privacy or ethical restrictions.
